# Histamine Increases Th2 Cytokine-Induced CCL18 Expression in Human M2 Macrophages

**DOI:** 10.3390/ijms222111648

**Published:** 2021-10-28

**Authors:** Susanne Mommert, Judith Tabea Schaper, Katrin Schaper-Gerhardt, Ralf Gutzmer, Thomas Werfel

**Affiliations:** 1 Division of Immunodermatology and Allergy Research, Department of Dermatology and Allergy, Hannover Medical School, 30625 Hannover, Germany; judith-schaper@web.de (J.T.S.); Schaper-Gerhardt.Katrin@mh-hannover.de (K.S.-G.); Ralf.Gutzmer@muehlenkreiskliniken.de (R.G.); Werfel.Thomas@mh-hannover.de (T.W.); 2 Department of Dermatology, Mühlenkreiskliniken AöR, Ruhr University Bochum Campus Minden, 32427 Minden, Germany

**Keywords:** macrophages, CCL18, atopic dermatitis, histamine

## Abstract

The chemokine CCL18 is produced in cells of the myelomonocytic lineage and represents one of the most highly expressed chemokines in lesional skin and serum of atopic dermatitis patients. We investigated the role of histamine in CCL18 production in human monocyte-derived M2 macrophages differentiated in the presence of M-CSF and activated with IL-4, IL-13 or with IL-10. Since expression and regulation of histamine H1 receptor (H1R), H2R and H4R by IL-4 and IL-13 on human M2 macrophages were described, we analyzed expression of the histamine receptors in response to IL-10 stimulation by quantitative RT-PCR. IL-10 upregulated H2R and downregulated H4R mRNA expression by trend in M2 macrophages. IL-10, but in a more pronounced manner, IL-4 and IL-13, also upregulated CCL18. Histamine increased the cytokine-induced upregulation of CCL18 mRNA expression by stimulating the H2R. This effect was stronger in IL-10-stimulated M2 macrophages where the upregulation of CCL18 was confirmed at the protein level by ELISA using selective histamine receptor agonist and antagonists. The histamine-induced CCL18 upregulation in IL-10-activated M2 macrophages was almost similar in cells obtained from atopic dermatitis patients compared to cells from healthy control persons. In summary, our data stress a new function of histamine showing upregulation of the Th2 cells attracting chemokine CCL18 in human, activated M2 macrophages. This may have an impact on the course of atopic dermatitis and for the development of new therapeutic interventions.

## 1. Introduction

The chemoattractant cytokine CCL18 belongs to (CC) chemokines that signal through seven-transmembrane-spanning, pertussis toxin-sensitive G-protein-coupled receptors (GPCRs) [1].

In 2013, Islam et al. detected CCR8 as a functional receptor for CCL18 that induces cell chemotaxis and calcium influx after activation by CCL18 in human CCR8 transfected cells [2]. Expression of CCR8 was described on cutaneous lymphocyte-associated antigen (CLA)^+^ skin homing T cells, on Th2 cells and regulatory T cells leading to the migration of these cells in the direction of CCL18. Migration of activated and highly differentiated mouse Th2 cells towards CCL18 has also been observed in adapted experimental settings, however, CCL18 is only present in humans and has no murine homologue [2].

Beyond its high expression in dendritic cells (DCs) [3], CCL18 is also secreted from blood myeloid cells, monocytes and from M2 macrophages [4]

Importantly, among a variety of chemokines, CCL18 represents the most abundant one in the skin of atopic dermatitis (AD) patients [4,5]. High induction of CCL18 in DCs was observed after exposure to allergens [6]. In parallel to an observed increase in CCL18-producing monocyte/macrophages and DCs in the skin, elevated concentrations of CCL18 were detected in serum samples of AD patients when compared to healthy individuals [4].

AD is a chronic inflammatory skin disease characterized by highly pruritic, eczematous skin lesions, structural abnormalities in the epidermis as well as by immune dysregulation. The complex disease is triggered by genetic and environmental susceptibility factors. Different clinical phenotypes of AD and an association with other allergic diseases have now been widely accepted [7].

Increased populations of subtypes of T cells, DCs and macrophages, mast cells and eosinophils constitute the cellular infiltrate in lesional AD skin [8,9]. In acutely and chronically inflamed AD skin, an increase in the number of macrophages when compared to nonlesional skin was detected by Kiekens et al. [10]. Skin macrophages are thought to be of blood monocyte origin, and to be involved in host defense and immunity. In the context of type 2 immune responses, such as allergic inflammation in AD skin lesions, macrophages are polarized to an alternatively activated phenotype. In particular, in response to IL-4 or IL-13, which are mainly produced by Th2 cells, mast cells, and basophils, the phenotype of M2 macrophages, expressing several characteristic markers that influence cells and tissues in their microenvironment, is strongly promoted [11,12].

In allergic situations, histamine is released from prestored granules by mast cells or basophils or after de novo synthesis from other immune cells in situ [13,14]. Human M2 macrophages express the histamine H1 receptor (H1R), H2R and H4R which are upregulated during their differentiation from monocytes. Histamine regulates via these receptors the expression of macrophage differentiation—or surface markers [15].

We showed recently that IL-4 upregulates the H2R and H4R mRNA expression, whereas IL-13 exclusively upregulates the H4R mRNA expression in human M2 macrophages [16].

Activation of M2 macrophages with IL-4 or IL-13 leads to overexpression of CCL18 [4,17]. Slight upregulation of CCL18 production in monocytes cultured for 6 days with IL-10 in synergy with IL-4, and especially pronounced in IL-10-stimulated immature DCs, was described by van Lieshout et al. [18].

In context of these observations, we investigated the influence of histamine as an amplifier of cutaneous inflammation and itch which is present in allergic skin inflammation [13] in regard to CCL18 production.

IL-4-, IL-13- and IL-10-activated human M2 macrophages were treated with histamine and with selective ligands for the H1R, H2R and H4R to investigate a possible role of histamine on the regulation of CCL18 production.

Our study demonstrates that Th2 cytokines and mediators which are released during allergic inflammation may produce a local microenvironment providing upregulation of CCL18 production, a chemokine known to be associated with the extent and severity of AD [4,19].

## 2. Results

### 2.1. Histamine Increases the IL-4- and the IL-13-Induced Upregulation of CCL18 mRNA Expresssion by Stimulating the H2R in Human M2 Macrophages

A potential influence of histamine, targeting different histamine receptors, on CCL18 production in activated M2 macrophages was investigated. We stimulated IL-4- or IL-13-activated M2 macrophages with histamine or agonists selective for H1R, H2R and H4R and analyzed the regulation of CCL18 mRNA expression.

**Figure 1:**
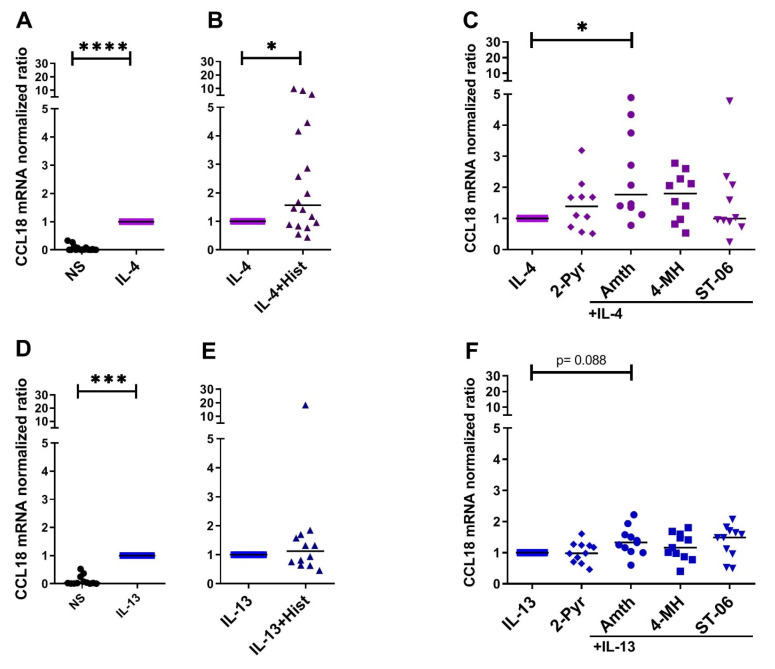
Histamine increases the IL-4- and the IL-13-induced upregulation of CCL18 mRNA expression by stimulating the H2R in human M2 macrophages. After differentiation in the presence of M-CSF, M2 macrophages were activated with (**A**–**C**), IL-4 (20 ng/mL) or (**D**–**F**), IL-13 (15 ng/mL) for 24 h. This was followed by a 24 h stimulation with (**B**), (**E**), histamine (Hist), (**C**,**F**), H1R agonist 2-pyridylethylamine (2-Pyr), H2R agonist amthamine (Amth), H2R/H4R agonist 4-methylhistamine (4-MH) and with the H4R agonist ST-1006 (ST-06) (all ligands 10 µM). CCL18 mRNA expression was detected by quantitative RT-PCR. The amount of the target mRNA relative to the amount of the reference gene, rps 20 mRNA in each stimulated sample, was normalized to the amount of the target mRNA relative to the amount of the reference gene in IL-4- or IL-13-stimulated samples and expressed as normalized ratio. Data are shown as individual values with medians. Significant differences, as determined by the Wilcoxon matched-pairs signed-rank test in (**A**,**B**,**D**,**E**) or by Friedman Dunn’s multiple comparison test in (**C**,**F**) are indicated as follows: * *p* < 0.05; *** *p* < 0.001; **** *p* < 0.0001; (**A**,**B**) (*n* = 18 independent donors and experiments); (**C**) (*n* = 10 independent donors and experiments); (**D**,**E**) (*n* = 12 independent donors and experiments); (**F**) (*n* = 11 independent donors and experiments); NS = nonstimulated.

Remarkably, we detected that histamine increases the IL-4-induced CCL18 mRNA upregulation by stimulation of the H2R in human M2 macrophages. Histamine (median 1.57-fold induction), the H2R agonist amthamine (median 1.77-fold induction) significantly and the H2R/H4R agonist 4-methylhistamine by trend (median 1.80-fold induction) potentiated the IL-4-induced CCL18 mRNA expression. Regarding the IL-13-stimulated macrophages, the H2R agonist amthamine (median 1.37-fold induction) upregulated the IL-13-induced CCL18 mRNA expression by trend. The H1R agonist 2-pyridylethylamine showed no effect (Figure 1A–F).

### 2.2. IL-10 Significantly Upregulates H2R but Downregulates H4R mRNA Expression by Trend in Human M2 Macrophages

**Figure 2:**
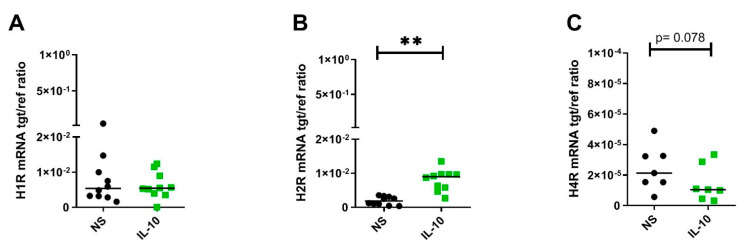
IL-10 significantly upregulates H2R but downregulates H4R mRNA expression by trend in human M2 macrophages. After differentiation in the presence of M-CSF, M2 macrophages were stimulated with IL-10 (10 ng/mL) for 48 h. A**,** H1R, B, H2R and C, H4R mRNA expression were detected by quantitative RT-PCR. The amount of the target mRNA relative to the amount of the reference gene, rps 20 mRNA in each stimulated sample, was expressed as target/reference (tgt/ref) ratio. Data are shown as individual values with medians. Significant differences, as determined by the Wilcoxon matched-pairs signed-rank test are indicated as follows: ** *p* < 0.01; (**A**,**B**) (*n* = 10 independent donors and experiments); (**C**) (*n* = 7 independent donors and experiments); NS = nonstimulated.

Beyond Th2 cytokines, the biogenic amine histamine is frequently detectable in enhanced concentrations in lesional skin of AD [13]. Monocyte-derived macrophages, which were found by immunohistochemistry or fluorescence in the dermal infiltrate of AD skin, [10] are known to express the histamine H1R, H2R, and H4R, which use histamine as a ligand [15,20]. Former studies by our research group observed elevated transcripts of H2R and H4R by IL-4 stimulation in eosinophils [21] and in M2 macrophages [16]. IL-13 exclusively upregulates the H4R mRNA expression in human eosinophils and M2 macrophages [16,21]. Since it has been assumed that beyond IL-4 or IL-13, Th2 cells secrete the anti-immune and anti-inflammatory cytokine IL-10 [22] we investigated whether IL-10 has functional effects on histamine receptor expression levels in human M2 macrophages. M-CSF-differentiated M2 macrophages were stimulated at day 8 with IL-10 (10 ng/mL) for 48 h. We could show that IL-10 also significantly upregulated the H2R mRNA expression. Surprisingly, we observed a trend towards downregulation of the H4R mRNA expression in M2 macrophages by IL-10 (Figure 2A–C).

### 2.3. CCL18 mRNA Expresion Is Upregulated by Th2 Cytokines IL-4, IL-13 and by IL-10 in Human M2 Macrophages

There is a differential mRNA expression of the histamine receptors in response to Th2 cytokines in human macrophages: IL-4 upregulates H2R and H4R, IL-13 exclusively upregulates H4R [16] and IL-10 upregulates H2R but downregulates H4R by trend in this study. Accordingly, we examined the ability of IL-4, IL-13 and IL-10 to regulate CCL18 expression in M2 macrophages obtained from the same donors.

**Figure 3:**
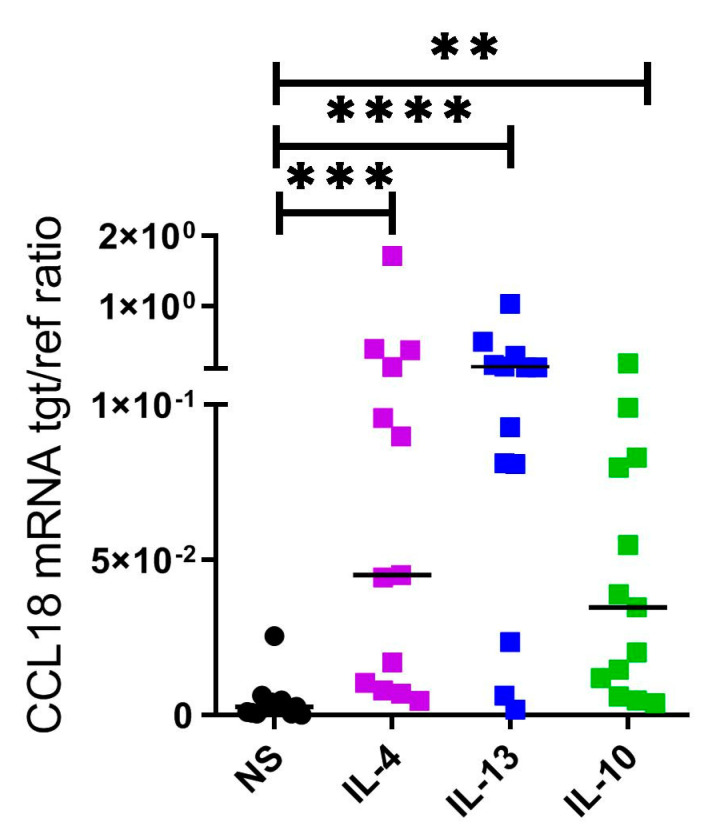
CCL18 mRNA expression is upregulated by Th2 cytokines IL-4, IL-13 and by IL-10 in human M2 macrophages. After differentiation in the presence of M-CSF, M2 macrophages from the same donors were activated with IL-4 (20 ng/mL), IL-13 (15 ng/mL) or IL-10 (10 ng/mL) for 48 h in parallel. CCL18 mRNA expression was detected by quantitative RT-PCR. The amount of the target mRNA relative to the amount of the reference gene, rps 20 mRNA in each stimulated sample, is depicted as target/reference (tgt/ref) ratio. Data are shown as individual values with medians. Significant differences, as determined by the Friedman Dunn´s multiple comparison test (NS as control column), are indicated as follows: ** *p* < 0.01; *** *p* < 0.001; **** *p* < 0.0001; (*n* = 13 independent donors and experiments); NS = nonstimulated.

In the presence of M-CSF, in vitro, differentiated monocyte-derived M2 macrophages were activated with the Th2 cytokines IL-4, IL-13 and IL-10 for 48 h. We detected in paired experiments an upregulation of CCL18 mRNA expression and protein secretion in response to all cytokines. Remarkably, upregulation of CCL18 expression was most pronounced after treatment with IL-4 and IL-13 when compared to treatment with IL-10 in a 48 h time period (Figure 3 and Supplementary Figure S1).

### 2.4. Histamine Increases CCL18 mRNA Expression by Stimulating the H2R in IL-10-Activated Human M2 Macrophages

**Figure 4:**
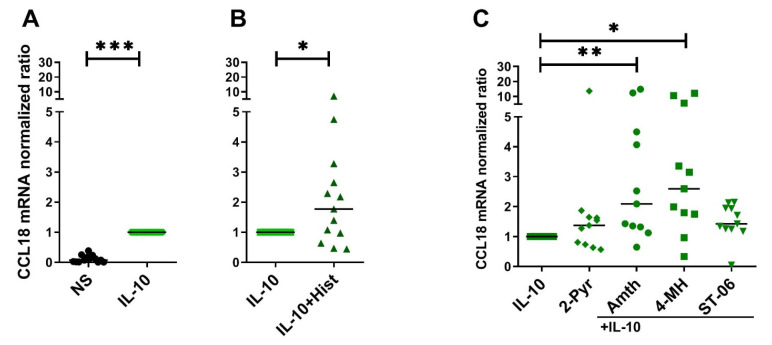
Histamine increases CCL18 mRNA expression by stimulating the H2R in IL-10-activated human M2 macrophages. After differentiation in the presence of M-CSF, M2 macrophages were activated with IL-10 (10 ng/mL) for 24 h. This was followed by a 24 h stimulation with histamine (Hist), H1R agonist 2-pyridylethylamine (2-Pyr), H2R agonist amthamine (Amth), H2R/H4R agonist 4-methylhistamine (4-MH) and with the H4R agonist ST-1006 (ST-06) (all ligands 10 µM). CCL18 mRNA expression was detected by quantitative RT-PCR. The amount of the target mRNA relative to the amount of the reference gene, rps 20 mRNA in each stimulated sample was normalized to the amount of the target mRNA relative to the amount of the reference gene in IL-10-stimulated samples and expressed as normalized ratio. Data are shown as individual values with medians. Significant differences, as determined by the Wilcoxon matched-pairs signed-rank test in (**A**,**B**) or by Friedman Dunn´s multiple comparison test in (**C**) are indicated as follows: * *p* < 0.05; ** *p* < 0.01; *** *p* < 0.001; (**A**,**B**) (*n* = 13 independent donors and experiments); (**C**) (*n* = 11 independent donors and experiments); NS = nonstimulated.

We assumed that the moderate increase in CCL18 mRNA expression and protein secretion by IL-10 compared to stronger effects of IL-4 or IL-13 (Figure 3 and Supplementary Figure S1) offers a higher possibility to detect the potentiating effects of histamine on CCL18 synthesis in IL-10-activated M2 macrophages. We stimulated IL-10-activated M2 macrophages with histamine or agonists selective for H1R, H2R and H4R and analyzed the regulation of CCL18 mRNA expression. We observed an upregulation of CCL18 mRNA expression in response to histamine (median 1.77-fold induction) by stimulating the H2R (amthamine, median 2.09-fold induction) and the H2R/H4R (4-methylhistamine, median 2.60-fold induction) (Figure 4A–C).

### 2.5. Histamine Increases CCL18 Protein Production by Stimulating the H2R in IL-10-Activated Human M2 Macrophages

After differentiation in the presence of M-CSF, we stimulated IL-4-, IL-13- or IL-10- activated M2 macrophages with histamine or agonists selective for H1R, H2R and H4R. Regulation of CCL18 protein secretion was investigated by ELISA.

**Figure 5:**
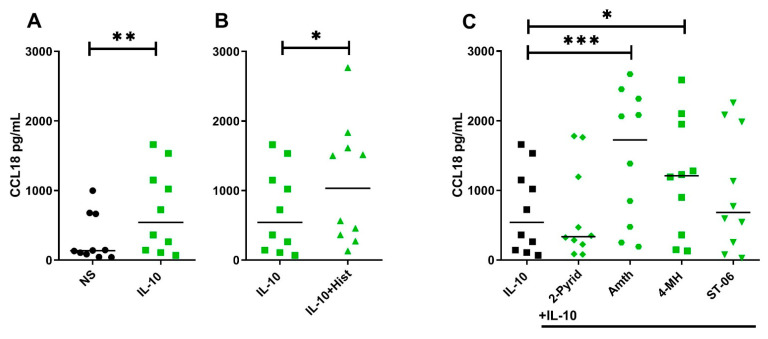
Histamine increases CCL18 protein production by stimulating the H2R in IL-10-activated human M2 macrophages. After differentiation in the presence of M-CSF, M2 macrophages were activated with IL-10 (10 ng/mL) for 24 h. This was followed by a 24 h stimulation with histamine (Hist), the H1R agonist 2-pyridylethylamine (2-Pyr), the H2R agonist amthamine (Amth), the H2R/H4R agonist 4-methylhistamine (4-MH) and the H4R agonist ST-1006 (ST-06) (all ligands 10 µM). CCL18 production was detected by ELISA. Data are shown as individual values with medians. Significant differences, as determined by the Wilcoxon matched-pairs signed-rank test in (**A**,**B**) or by Friedman Dunn´s multiple comparison test in (**C**) are indicated as follows: * *p* < 0.05; ** *p* < 0.01; *** *p* < 0.001; (**A**–**C**) (*n* = 10 experiments); NS = nonstimulated.

We observed no or only marginal effects of histamine and receptor-selective agonists on the CCL18 protein production in IL-4- or IL-13-activated M2 macrophages (data not shown). Our hypothesis that histamine effects are more likely to be detected in IL-10-stimulated M2 macrophages was supported by analyzing CCL18 production at the protein level by ELISA technique. In accordance with the clear picture showing regulation of CCL18 mRNA expression by histamine stimulating the H2R in IL-10-activated M2 macrophages, CCL18 protein production was also potentiated in response to histamine (median 1.91-fold induction), to the H2R agonist amthamine (median 3.18-fold induction), to the H2R/H4R agonist 4-methylhistamine (median 2.23-fold induction) and by trend in response to the H4R agonist ST-1006 in these cells (Figure 5A–C).

### 2.6. The Potentiating Effect of Histamine on CCL18 Protein Production in IL-10-Activated M2 Macrophages Was Inhibited by Preincubating the Cells with the Selective H2R Antagonist

**Figure 6:**
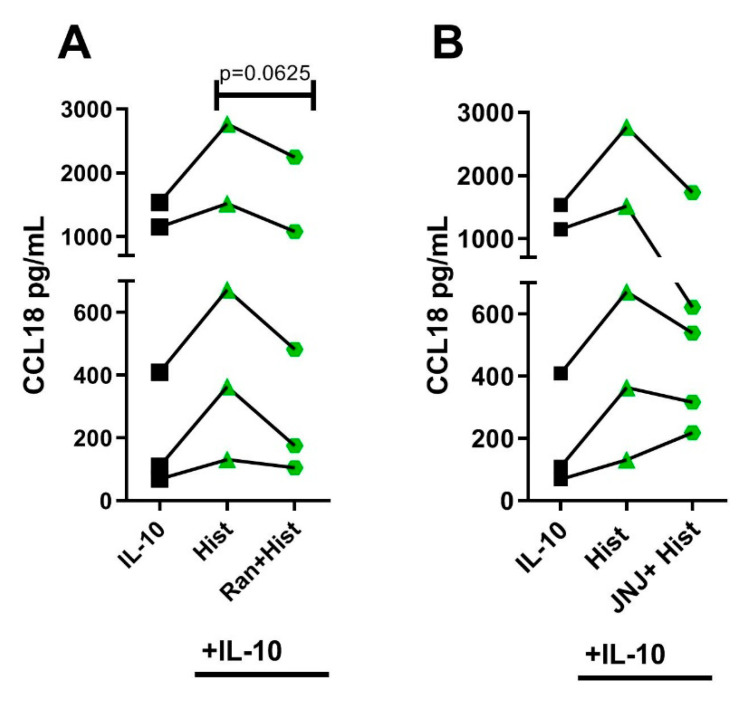
The potentiating effect of histamine on CCL18 protein production in IL-10-activated M2 macrophages was inhibited by preincubating the cells with selective antagonists. After differentiation in the presence of M-CSF, M2 macrophages were activated with IL-10 (10 ng/mL) for 24 h. This was followed by a 24 h stimulation with histamine (Hist). Only experiments with a robust upregulation of CCL18 by histamine were included in the blocking experiments. (**A**) the H2R and (**B**) the H4R were blocked with their selective antagonists 30 min before histamine stimulation: H2R: Ranitidine (Ran); H4R: JNJ7777120 (JNJ) (all ligands 10 µM). CCL18 production was detected by ELISA. Data are shown as individual values. Statistics were determined by the Wilcoxon matched-pairs signed-rank test. (*n* = 5 experiments); NS = nonstimulated.

To further assess whether the IL-10-induced upregulation of CCL18 increased by histamine is mediated by stimulating the H2R or H4R, M2 macrophages were stimulated with IL-10 for 24 h and with histamine for additional 24 h. The H2R and H4R were blocked by their selective antagonists 30 min before histamine stimulation. The concentration of CCL18 was measured by ELISA. We observed that the effect of histamine was inhibited by the selective H2R antagonist ranitidine for all donors, whereas the H4R antagonist JNJ7777120 only inhibited the effect of histamine in three out of five donors, which suggests a predominant role of the H2R in the regulation of CCL18 (Figure 6A,B).

### 2.7. Upregulation of CCL18 mRNA Expression in Response to H2R Stimulation Is More Pronounced in Human IL-10-Activatd M2 Macrophages from Healthy Control Persons When Compared to Cells from AD Patients

Higher levels of CCL18 were previously found in serum from AD patients when compared to healthy control persons. Expression of CCL18 was detected in close association with mononuclear cell infiltrates in the skin of AD patients in that study. These elevated CCL18 levels were present in the skin of AD patients but absent in the skin from healthy control persons [4].

**Figure 7:**
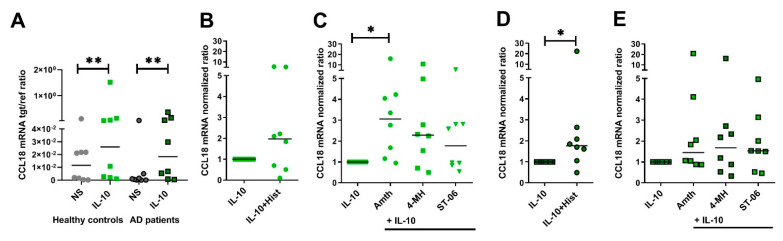
Upregulation of CCL18 mRNA expression in response to H2R stimulation is more pronounced in human IL-10-activated M2 macrophages from healthy control persons when compared to cells from atopic dermatitis (AD) patients. After differentiation in the presence of M-CSF, M2 macrophages obtained from healthy control persons (**B**,**C**) and from AD patients (**D**,**E**) were activated with IL-10 (10 ng/mL) for 24 h. This was followed by a 24 h stimulation with histamine (Hist), H2R agonist amthamine (Amth), the H2R/H4R agonist 4-methylhistamine (4-MH) and with the H4R agonist ST-1006 (ST-06) (all ligands 10 µM). CCL18 mRNA expression was detected by quantitative RT-PCR. (**A**), constitutive expression of CCL18 in nonstimulated and in IL-10-activiated M2 macrophages from healthy control persons compared to cells from AD patients shown as target/reference (tgt/ref) ratio. (**B**,**C**), M2 macrophages from healthy control persons, (**D**,**E**), M2 macrophages from AD patients. The amount of the target mRNA relative to the amount of the reference gene, rps 20 mRNA in each stimulated sample, was normalized to the amount of the target mRNA relative to the amount of the reference gene in IL-10-stimulated samples and expressed as normalized ratio. Data are shown as individual values with medians. Significant differences, as determined by the Wilcoxon matched-pairs signed-rank test in (**A**,**D**) or by Friedman Dunn´s multiple comparison test in (**C**) are indicated as follows: * *p* < 0.05; ** *p* < 0.01; (**A**–**E**) (*n* = 8 experiments); NS = nonstimulated.

To investigate the effects of histamine on IL-10-induced CCL18 mRNA expression in cells from healthy control persons versus cells from AD patients, human monocyte-derived M2 macrophages obtained from both groups were stimulated for 24 h with IL-10 and after 24 h with histamine and selective agonists targeting the H1R, H2R and H4R for an additional 24 h. IL-10 increased CCL18 mRNA expression both in control and AD groups. However, we found no significant difference of CCL18 mRNA expression when comparing cells from healthy control persons to AD patients with the Mann–Whitney test (Figure 7A).

Histamine upregulated CCL18 mRNA expression in both groups (histamine median 1.97-fold in healthy control persons versus 1.77-fold induction in AD patients). However, due to the high donor-dependent variability in the group of healthy control persons, the upregulation of IL-10-induced CCL18 mRNA expression in response to histamine was significant only in cells from AD patients (Figure 7B,D). Finally, we observed a significant upregulation of CCL18 mRNA expression in M2 macrophages from healthy control persons by stimulating the H2R (amthamine median 3.06-fold induction) and by trend by stimulating H2R/H4R (4-MH median 2.29-fold induction). In comparison to cells from AD patients, upregulation of CCL18 mRNA expression was not regulated by these agonists (amthamine median 1.45-fold induction, 4-MH median 1.68-fold induction) Figure 7C,E).

## 3. Discussion

In contrast to normal or psoriatic skin, it was demonstrated that the expression of the T-cell-attracting chemokine CCL18 is upregulated by tissue-resident antigen-presenting cells in response to multiple environmental stimulations in AD skin, supporting the important role of CCL18 in the organization of innate and adaptive immune responses in the disease [4,19,23].

A potential relevance of monocyte-derived cells beyond T cells in AD was provided by observations of Kiekens et al., where an increase in macrophage numbers, identified by the macrophage differentiation marker CD68 and expression of mannose receptors, was detected in a model of acute AD as well as in skin biopsies of chronic, inflamed, lesional AD skin by immunohistochemistry. Interestingly, the numbers of dendritic cells remained unchanged [10]. The inflammatory skin disease is characterized by cutaneous hyperreactivity to allergens and by a microenvironment in the skin enriched with Th2 cytokines, such as IL-4 and IL-13, and histamine released from activated immune cells [7,8,9].

We hypothesized that the highly abundant expression of CCL18 in AD skin is related to the contact of IL-4-, IL-13- or IL-10-activated macrophages with histamine.

In order to directly compare the efficacy of these cytokines in upregulating CCL18 expression in macrophages, we investigated the CCL18 mRNA and protein production in human M-CSF-differentiated M2 macrophages after IL-4, IL-13 or IL-10 stimulation in parallel in cells obtained from the same donors. In this approach, shown here for the first time, we observed that the upregulation of CCL18 expression was most pronounced after IL-4 and IL-13 stimulation when compared to IL-10 stimulation. The upregulation of CCL18 expression by these cytokines has been described in different ways in older and more recent literature:

In monocyte-derived DCs cultured in GM-CSF and IL-4, it was shown that the anti-inflammatory cytokine IL-10 strongly induces CCL18 production, whereas stimulation of DCs after maturation of 6 days with TNF-α, IL-1β, IL-13, IL-15, IL-17, IL-18 and IFN-γ had no effect [18]. Van Lieshout et al. showed in contrast to us that IL-10 is only able to enhance CCL18 upregulation in synergy with IL-4 or IL-13 in monocytes, and IL-10 alone had only minimal effects on CCL18 production in monocytes or macrophages in their study [18]. IL-10 has been shown to promote the development of a Th2 response and to suppress a Th1 response. Th2 cells produce respectable amounts of antigen-specific IL-10 [22]. IL-10 is overexpressed in the skin of AD patients and has an essential function for skin infiltration with eosinophils in a mouse model of atopic dermatitis [24]. In this regard we classify in this work IL-10 as a Th2-associated cytokine. We showed that macrophages respond to IL-10 with CCL18 production but produce smaller amounts of CCL18 when compared to stimulation with IL-4 or IL-13. Vulcano et al. also detected IL-10-induced CCL18 production in blood myeloid cells [25].

In a recent publication, upregulation of CCL18 by IL-4 was described for macrophages derived from the monocytic cell line THP-1 and for human primary macrophages at mRNA level [26]. Lescoat et al. showed that JAK inhibitors are able to limit M2a activation of macrophages by IL-4 and IL-13. This was demonstrated by the downregulation of the M2a-associated surface marker CD206 and by the prevention of IL-4- and IL-13-induced secretion of CCL18 [17].

In AD, histamine is released from preformed stores in mast cells and basophils and by spontaneous secretion from other immune cells [14] and therefore is also considered as a marker of the molecular signature of AD skin.

Since there are currently no specific antibodies available to detect the histamine receptor expression in human immune cells [27,28] we evaluated the expression of H1R, H2R and H4R by quantitative PCR only.

In our previous studies we detected the mRNA expression of H1R, H2R, H4R in human M2 macrophages [15] as well as upregulation of H2R and H4R by IL-4 or IL-13 in M2 macrophages [16] and in human eosinophils [21]. 

Effects of IL-10 on histamine receptor expression levels on human macrophages have not been described so far. Here, we could show for the first time that IL-10 significantly upregulates the H2R expression, which was previously described after IL-4 stimulation [16]. In contrast to IL-4 and IL-13 [16], IL-10 seemed to downregulate the H4R in our experiments in M2 macrophages.

The main producers of IL-10 besides T cells, monocytes and DCs, are regulatory macrophages and also mast cells. The cytokine is known to limit a broad spectrum of inflammatory activities by inhibiting antigen presentation, upregulation of costimulatory molecules and mediate the release from DCs and macrophages [29,30]. Upregulation of H2R expression in combination with downregulation of H4R in M-CSF-differentiated macrophages occurs as a novel, homeostatic mechanism to prevent uncontrolled inflammation in response to IL-10. Activation of the H2R is strongly associated with immunoregulatory effects which can be intensified by upregulation of the receptor. This is supported by observations where histamine, released by microbes, reduces the LPS response via H2R in THP-1 cells [31] as well as by the H2R-mediated inhibition of TLR-induced upregulation of cytokines of the IL-12 family in monocytes, DCs [32] and plasmacytoid dendritic cells [33].

However, we observed that histamine increases the IL-4-and IL-10-induced upregulation of CCL18 expression by stimulating the H2R which was most pronounced in IL-10-activated M-CSF-differentiated M2 macrophages. We speculate that the histamine effect was partially masked by the strong induction of CCL18 in IL-4- or IL-13-activated macrophages, which was not the case in IL-10-stimulated cells, showing only a moderate upregulation of CCL18 expression.

Beyond CCL18, CCL17 mRNA transcripts are also highly abundant in lesional skin biopsy specimens from patients with moderate-to-severe AD [34]. In a previous work we found an upregulation of CCL17 expression, a well-known potent biomarker of AD, by histamine via H2R in M2 macrophages [35]. We observed no noteworthy, enhanced effects of histamine or of receptor-selective agonists on CCL18 expression when we compared macrophages from AD patients versus cells from healthy control persons. This was in accordance with our previous study, where the expression profile of CCL17 in response to histamine in M2 macrophages was almost equal in respective groups [35]. We suppose that patients suffering from AD have a general systemic Th2 background, and immune cells have a more frequent contact with allergic mediators that may promote an adaption to an inflammatory microenvironment. This may lead to unresponsiveness to further stimulation of the cells.

In conclusion, we provide evidence that increased numbers of macrophages and a microenvironment in the skin consisting of key molecules for AD such as Th2 cytokines and histamine may contribute to CCL18 expression, which is known to be highly upregulated in AD skin and in serum samples from AD patients [4,5].

High expression levels of CCL18 correlate with disease severity [36]. However, the role of CCL18 on immune cells in the inflammatory environment is ambivalent.

On one hand, CCL18 shows proinflammatory capacity through its chemotactic activity mainly for T cells [4], through the maturation of monocytes to macrophages which produce inflammatory mediators [37], and through activation of basophils, which could also infiltrate skin lesions [38], to release histamine in response to CCL18 [39]. The chemokine CCL18 was also suggested to promote pulmonary fibrosis [40].

**Figure 8:**
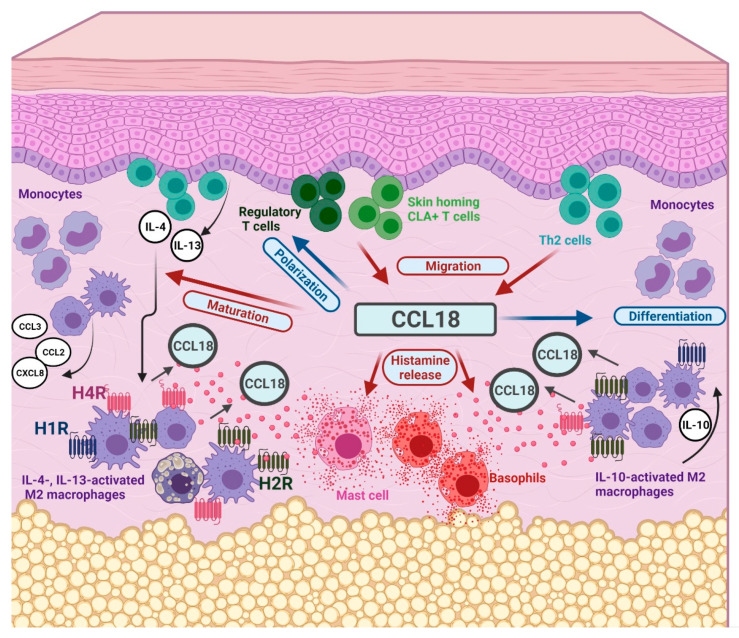
Ambivalent function of the chemokine CCL18 between inflammation and its resolution. CCL18 is produced by antigen-presenting cells and macrophages in the skin after activation with Th2 cytokines IL-4, IL-13 and IL-10. Environmental exposure to histamine increases the cytokine-induced release of CCL18 from human M2 macrophages. Beyond its chemotactic properties mainly for Th2 cells, skin-homing, cutaneous lymphocyte-associated antigen (CLA)+ T cells and regulatory T cells, CCL18 has direct immunomodulatory effects on the cells constituting the inflammatory infiltrate in AD skin. CCL18 induces maturation of monocytes to macrophages which produce inflammatory chemokines and fosters histamine release from basophils. The polarization of human CD4+ T cells into regulatory Foxp3+ T cells and its ability to differentiate monocytes to tolerogenic IL-10 secreting cells show more the regulatory effects of the chemokine CCL18 (Simplified overview, created with BioRender.com assess on 25/10/2021). Tolerogenic functions of CCL18 are indicated by blue arrows and in blue shaped boxes, the more proinflammatory functions of CCL18 are indicated by red arrows and in red shaped boxes, H1R = histamine H1 receptor, H2R = histamine H2 receptor, H4R = histamine H4 receptor.

On the other hand, CCL18, as a competitive antagonist of CCR3, abrogates migration mainly of eosinophils in response to CCL11 or CCL13 [41], differentiates monocytes to tolerogenic IL-10 secreting cells [3] and polarizes human CD4+ T cells into regulatory Foxp3+ T cells [42] (Figure 8).

These facts may change the focus of research and show that CCL18 can also lead to the resolution of inflammation and can thus influence the course of a pathological event in one direction or in the other at a critical point.

The blockade of the IL-4/IL-13 pathways or the therapeutic use of histamine receptor antagonists may, via the regulation of the expression of CCL18, lead to a shift from an inflammatory to a more homeostatic situation.

## 4. Materials and Methods

### 4.1. Isolation of Monocyte Derived Macrophages

Residual blood samples from platelet (PLT) apheresis disposables used for routine PLT collection and of regular anonymous healthy donors (buffy coats) served as source material for the isolation of human peripheral blood mononuclear cells (PBMCs). We measured total serum IgE levels in the serum of buffy coats by ImmunoCap Thermofisher Scientific (Freiburg, Germany). Slightly increased total IgE values were detected only in two samples. These samples showed no deviations from the other samples in the subsequent PCR and ELISA analysis. In the group of IL-10-stimulated macrophages, total IgE values were in the normal range. Macrophages were differentiated from buffy coats for the experiments which are depicted in Figure 1, Figure 2, Figure 3, Figure 4, Figure 5 and Figure 6.

Healthy control persons (Caucasian, *n* = 8, 5 females and 3 males mean age 35.6 ± 9.9 years) and patients with moderate-to-severe extrinsic atopic dermatitis (AD) which received no systemic treatment (Caucasian, *n* = 8, 3 females and 5 males mean age 39.0 ±16.1 years) were recruited from the Department of Dermatology and Allergy, Hannover Medical School, Hannover, Germany. AD was diagnosed according to the criteria of Hanifin and Rajka [43]. Venous blood samples were taken from both groups. Macrophages were differentiated from these cells for the experiments which are depicted in Figure 7.

PBMCs were separated by density gradient centrifugation on lymphoprep (Fresenius Kabi Norge AS, Oslo, Norway). With a seeding density of 1 × 10^6^ cells pro well, PBMCs were plated in a 24-well plate in Iscoves Medium supplemented with Human Serum AB male (PAN Biotech GmbH, Aidenbach, Germany) (2.5% *v*/*v*). To attach the monocytes, cells were incubated for 2 h at 5% CO_2_ and 37 °C. Nonadherent cells were removed by vigorously washing of adherent cells three times with PBS. An appropriate amount of RPMI 1640, supplemented with 2 mM l-glutamine, 100 mg/mL penicillin/streptomycin, 12 mM Hepes, and 5% *v*/*v* Human Serum AB male (PAN Biotech GmbH, Aidenbach, Germany; all other media components from Biochrom, Berlin, Germany) and 10 ng/mL macrophage colony-stimulating factor (M-CSF) (R&D, San Diego, CA, USA) was added.

### 4.2. Cell Culture, Differentiation of M2 Macrophages

Cells were cultured with M-CSF during the differentiation process to M2 macrophages.

At day 5, another 50% by volume of fresh medium containing M-CSF was added. At day 8, the medium was completely changed. After 10 days, M2 macrophages appear as adherent cells with a prominent nucleus and multiple pseudopodia.

The differentiation process was controlled by analyzing the expression levels of macrophage differentiation marker CD68 (data not shown). In our previous publication we could show that the M-CSF-differentiated macrophages according to our protocol express the costimulatory molecule CD86 and the M2 markers CD206 and CD163. CD80 was very low or not expressed [44].

### 4.3. Histamine Receptor Ligands

The following histamine receptor ligands were used in this study: Histamine (ALK-Abello, Madrid, Spain) as agonist for all histamine receptors; 2-pyridylethylamine (Tocris Bioscience, Bristol, UK) as selective H1R agonist; amthamine (Tocris Bioscience, Bristol, UK) as selective H2R agonist; H2R and H4R agonist 4-methylhistamine (4MH) (Tocris Bioscience, Bristol, UK); ST-1006 as selective H4R agonist [45] (Institute of Pharmaceutical and Medicinal Chemistry, Heinrich Heine University, Duesseldorf, Germany); ranitidine (Sigma Aldrich, Deisenhofen, Germany) as selective H2R antagonist; JNJ7777120 as selective H4R antagonist (Sigma Aldrich, Deisenhofen, Germany); all histamine receptor ligands were used at a concentration of 10 µM. In extensive previous studies we could show that the concentration of 10 µM is optimal to demonstrate and reproduce robust histamine-receptor ligand-mediated effects [46].

### 4.4. Stimulation of M2 Macrophages

M2 macrophages were stimulated with IL-4 (20 ng/mL), IL-13 (15 ng/mL (R&D Systems, Wiesbaden, Germany)) or IL-10 (10 ng/mL (R&D Systems, Wiesbaden, Germany)) for 48 h. The time duration of 48 h was chosen after performing time kinetics (Supplementary Figure S1). After 48 h there was a significant upregulation of CCL18 production in response to IL-4, IL-13 and also to IL-10.

IL-4-, IL-13- or IL-10-activated M2 macrophages (24 h) were stimulated with histamine, 2-pyridylethylamine (H1R agonist), amthamine (H2R agonist), 4-methylhistamine (4-MH) (H2R/H4R agonist) or ST-1006 (H4R agonist) (10 µM) for 24 h. This results in a total incubation for IL-4-, IL-13- or IL-10 of 48 h. RNA was stabilized in lysis buffer and cell-free supernatants were collected.

CCL18 mRNA and protein expression were analyzed by quantitative Real Time (RT)-PCR and ELISA, respectively. The ELISA was performed according to the manufacturer’s instructions (R&D Systems, Wiesbaden, Germany).

For blocking experiments, IL-10-activated M2 macrophages were preincubated with the selective antagonists for the H2R (ranitidine) or for the H4R (JNJ7777120) 30 min before stimulation.

### 4.5. Quantitative Real Time (RT)-PCR

Total RNA was isolated using the RNeasy kit (Qiagen, Hilden, Germany) according to the manufacturer’s instructions. The cDNA was synthesized by reverse transcription (QuantiTect reverse transcription kit, Qiagen, Hilden, Germany). Quantitative RT-PCR was performed according to the MIQE guidelines with Quantitect^®^ primer assays for CCL18 (QT00000553), H1R (QT00199857), H2R (QT00210378), H4R (QT00032326), H4R 390 (1912485, 1912486) (TIB Molbiol Syntheselabor, Berlin, Germany) and rps 20 (ribosomal protein S20) (QT00079247) using SYBR^®^ Green according to the manufacturer’s instructions (Qiagen, Hilden, Germany) using the LightCycler (LC) 480.

The amount of the target mRNA relative to the amount of the reference gene mRNA, ribosomal protein S20 (rps 20), in the same sample was calculated using the comparative Ct method also known as the [delta] [delta] Ct method provided by the Software LC 480 (Roche Molecular Biochemicals, Mannheim, Germany).

### 4.6. Statistics

For statistical analyses, the software GraphPad Prism Version 8.0 was used (GraphPad software, San Diego, CA, USA). First, we performed methods to test the normal Gaussian distribution of the data. In all our experiments, due to the individual variations of the data, the normality tests failed. The nonparametric test Wilcoxon matched-pairs signed-rank test was used to test whether the medians of two independent samples are different. Friedman Dunn’s Multiple Comparison test was used for group comparisons. The medians are shown in the graphs. A *p*-value < 0.05 was regarded as statistically significant (*p* < 0.05 was labelled with *, *p* < 0.01 was labelled with **, *p* < 0.001 was labelled with ***, *p* < 0.0001 was labelled with ****).

## Data Availability

The data that support the findings of this study are available from the corresponding author upon reasonable request.

## Supplementary Materials

The following are available online at https://www.mdpi.com/article/10.3390/ijms222111648/s1.
