# Supplementary material for: Histamine Increases Th2 Cytokine-Induced CCL18 Expression in Human M2 Macrophages
Source: Int J Mol Sci. 2021 Oct 28;22(21):11648. doi: 10.3390/ijms222111648 (PMC8584115; doi:10.3390/ijms222111648)
Supplement: Supplementary file 1 [file ijms-22-11648-s001.zip › ijms-1368635-supplementary.pdf]

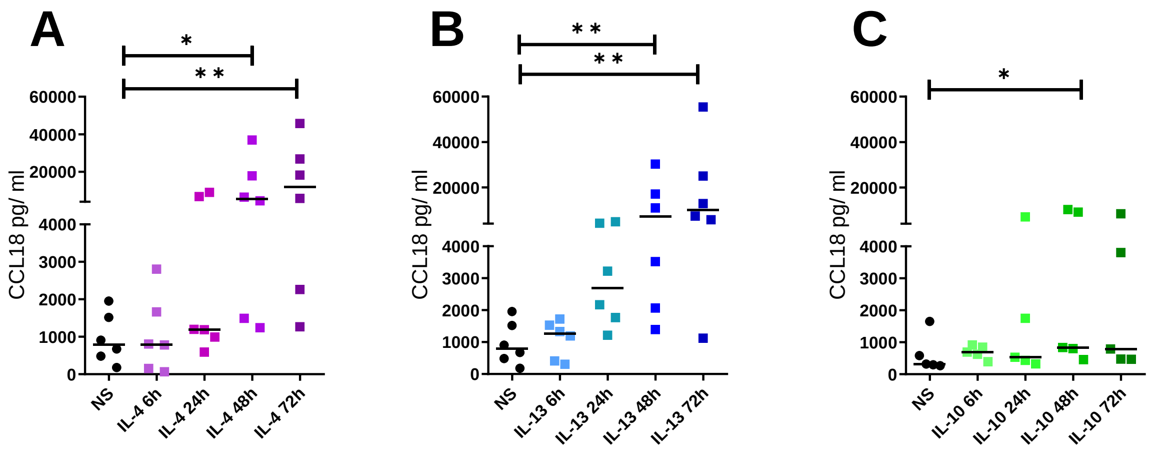

Supplementary Figure S1: CCL18 protein expression is time dependent up-regulated by IL-4, IL-13 and by IL-10 in human M2 macrophages.

After differentiation in the presence of M-CSF, M2 macrophages were activated with A, IL-4 (20 ng/ml) or B, IL-13 (15 ng/ml) or C, IL-10 (10ng/ml) for different time-periods as indicated. CCL18 protein concentration was analysed by ELISA. Data shown are individual values with medians. Significant differences, as determined by Friedman Dunn's multiple comparison test are indicated as follows: *P < .05; **P < .01; A-C (n = 6 independent donors and experiments), NS = non-stimulated.
